# Supplementary material for: Evaluation of PSA and PSA Density in a Multiparametric Magnetic Resonance Imaging-Directed Diagnostic Pathway for Suspected Prostate Cancer: The INNOVATE Trial
Source: Cancers (Basel). 2021 Apr 20;13(8):1985. doi: 10.3390/cancers13081985 (PMC8074769; doi:10.3390/cancers13081985)
Supplement: Supplementary file 1 [file cancers-13-01985-s001.zip › cancers-1153951-SI.pdf]

cancers

Supplementary Materials: Evaluation of PSA and PSA Density in a Multiparametric Magnetic Resonance Imaging-Directed Diagnostic Pathway for Suspected Prostate Cancer: The INNOVATE Trial

Hayley Pye, Saurabh Singh, Joseph M. Norris, Lina M. Carmona Echeverria, Vasilis Stavrinides, Alistair Grey, Eoin Dinneen, Elly Pilavachi, Joey Clemente, Susan Heavey, Urszula Stopka-Farooqui, Benjamin S. Simpson, Elisenda Bonet-Carne, Dominic Patel, Peter Barker, Keith Burling, Nicola Stevens, Tony Ng, Eleftheria Panagiotaki, David Hawkes, Daniel C. Alexander, Manuel Rodriguez-Justo, Aiman Haider, Alex Freeman, Alex Kirkham, David Atkinson, Clare Allen, Greg Shaw, Teresita Beeston, Mrishta Brizmohun Appayya, Arash Latifoltojar, Edward W. Johnston, Mark Emberton, Caroline M. Moore, Hashim U. Ahmed, Shonit Punwani and Hayley C. Whitaker

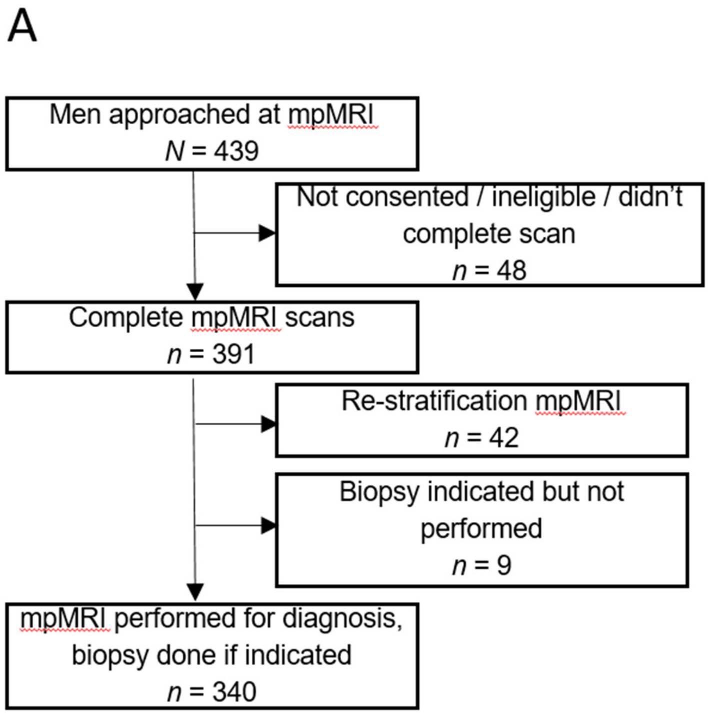

B

Inclusion criteria
1. Biopsy naive men presenting to our institution with a clinical suspicion of prostate cancer
2. Men referred to our center for prostate mp-MRI following biopsy elsewhere
**Exclusion Criteria**
1. Men unable to have a MRI scan, or in whom artifact would reduce quality of MRI
2. Men unable to given informed consent,
3. Previous treatment (prostatectomy, radiotherapy, brachytherapy) of prostate cancer,
4. On-going hormonal treatment for prostate cancer
5. Previous biopsy within 6 months of scheduled mp-MRI

Figure S1. (A) Flow diagram for patient recruitment in the INNOVATE trial. *n* = number of patients; mpMRI = multiparametric magnetic resonance imaging. (B) Table detailing the inclusion and exclusion criteria for the full trial.

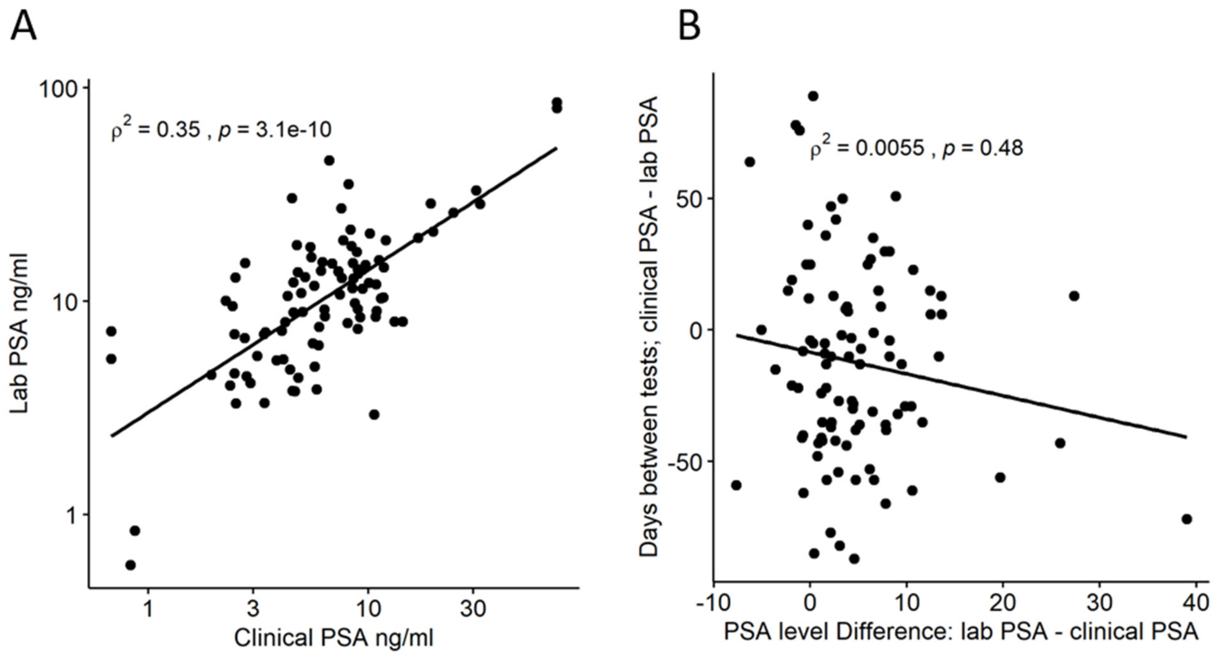

		Two sided pearson's product-moment correlation			
	Comparison	estimate	statistic	p.value	Rho(ρ) squared
A	Log2 lab PSA vs log2 Clinical PSA	0.590017	58578.41	3.14E-10	0.34812
B	The difference in both PSA results vs time between the two tests	-0.07405	153460.8	0.475716	0.005484

Figure S2. Total serum PSA re-tested in 95 patients. New serum sample taken at mpMRI, processed in our lab and sent for analysis at an external clinical testing centre. (A) Correlation between this new 'lab' PSA and the reported 'clinical' PSA from patient notes. (B) Correlation between the difference in ng/ml between the two tests and the time in days between the two tests. Displayed with linear regression line. Full statistical output of the Spearman's rank correlation below in a table.

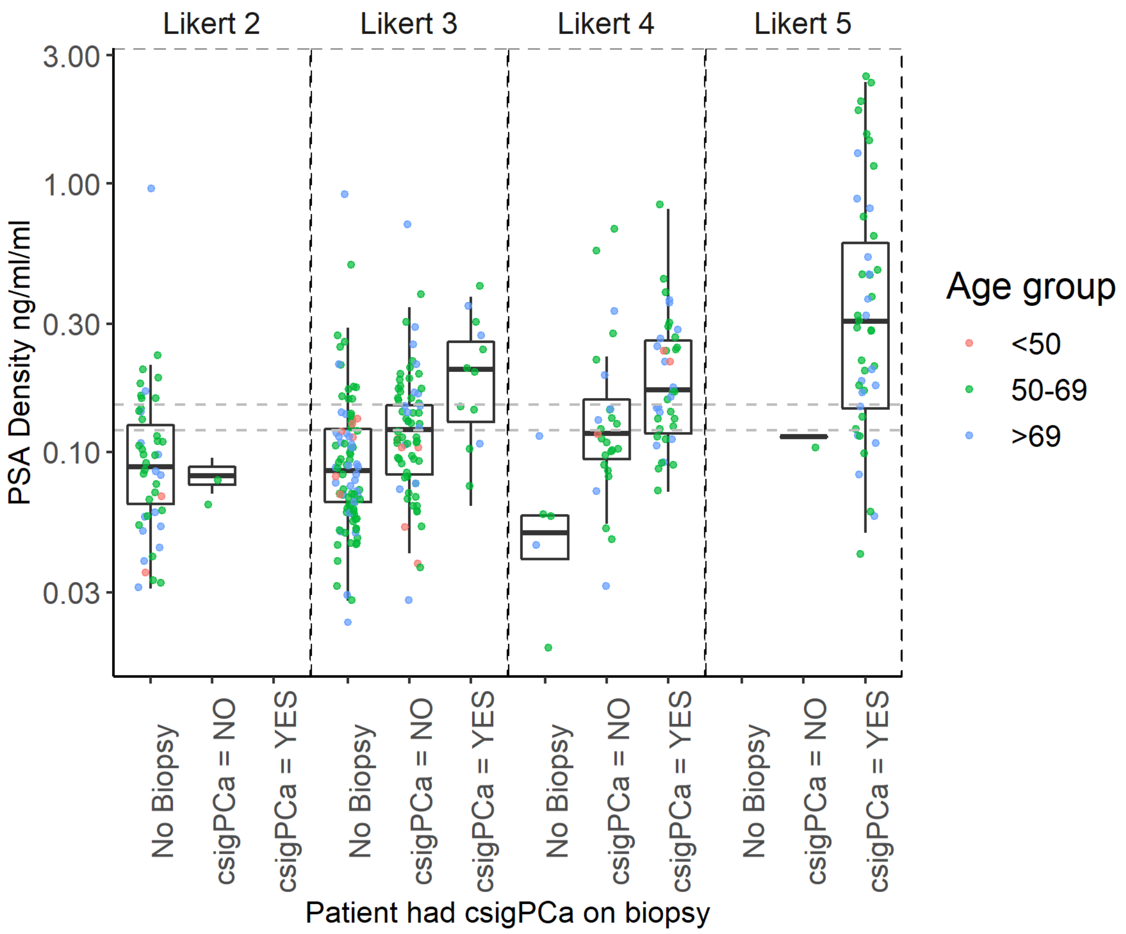

Two sided Wilcoxon Rank Sum Test ('Mann-Whitney' test)	Median (IQR)	Median difference	test statistic	p.value	Symbol
PSA Density (ng/ml/ml) Within Likert 3	csigPCa=NO: 0.12 (0.08, 0.15)<br>csigPCa=YES: 0.20 (0.13, 0.26)	0.08	236	0.0158	*
PSA Density (ng/ml/ml) Within Likert 4	csigPCa=NO: 0.12 (0.09, 0.16)<br>csigPCa=YES: 0.17 (0.12, 0.26)	0.05	342	0.0281	*

Figure S3. PSA density of all patients, grouped as 1. without prostate biopsy, 2. without csigPCa on subsequent biopsy and 3. with csigPCa on biopsy. Clinically significant prostate cancer (csigPCa) as defined as overall Gleason ≥3+4 of any length OR maximum cancer core length (MCCL) ≥4mm of any grade. Stratified by Likert score. Raw values for each patient shown as data points overlaying a boxplot displaying central line as median, lower and upper hinges as 25th and 75th percentiles (Q1 and Q3) and upper and lower whiskers as the last value no further than 1.5 * IQR from the hinge. Points coloured by patient age group and horizontal lines grey dashed lines at PSA density values of 0.12 and 0.15 ng/ml/ml. (Asterix denotes significance level in testing; * = P ≤ 0.05, ** = P ≤ 0.01, *** = P ≤ 0.001, **** =P ≤ 0.0001). Full statistical output in table below. Not all groups compared.

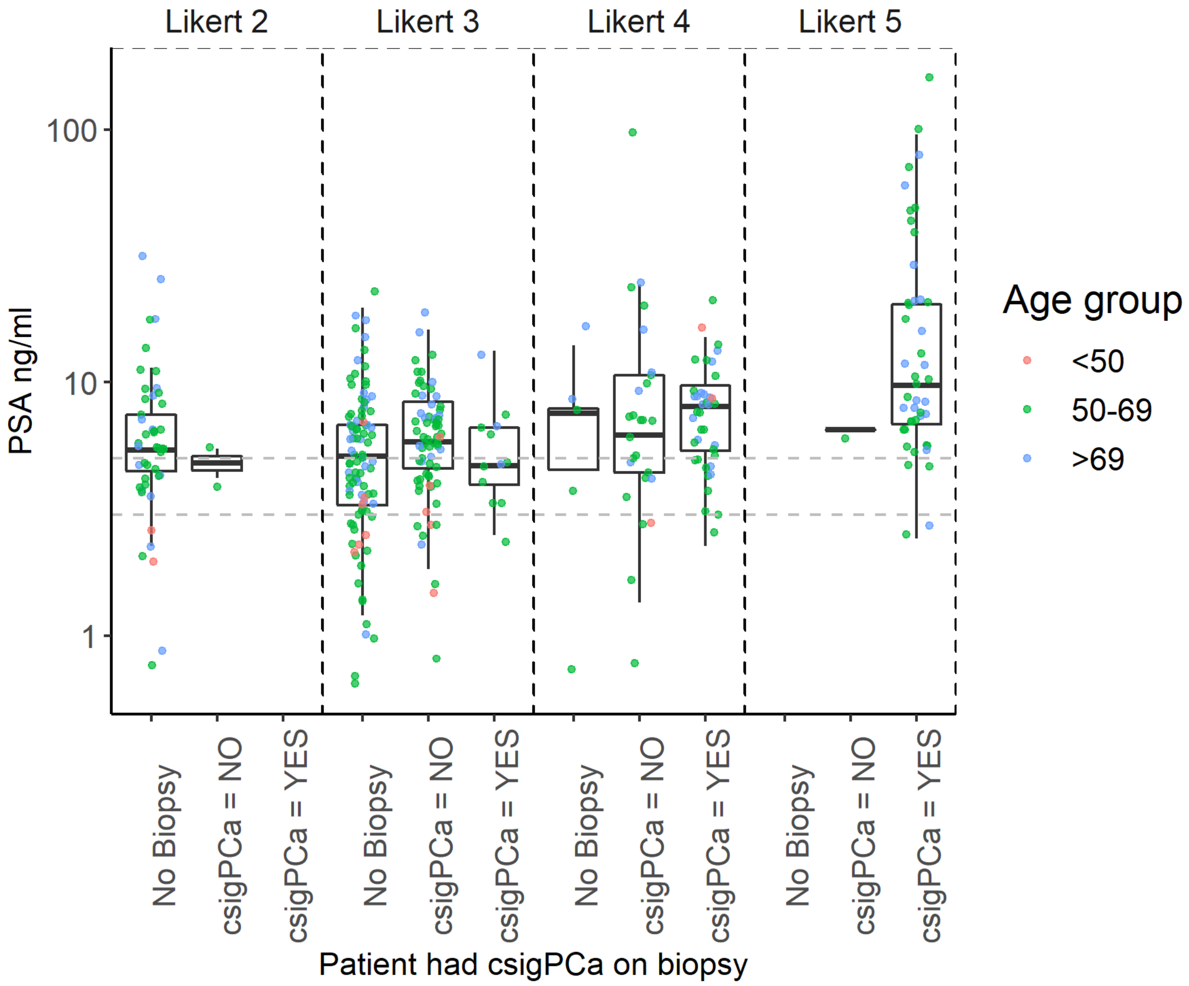

Two sided Wilcoxon Rank Sum Test ('Mann-Whitney' test)	Median (IQR)	Median difference	test statistic	p.value	Symbol
PSA (ng/ml) Within Likert 3	csigPCa=NO: 5.8 (4.6, 8.4) csigPCa=YES: 5 (4, 7)	0.8	502	0.285	ns
PSA (ng/ml) Within Likert 4	csigPCa=NO: 6.2 (4.4, 10.7) csigPCa=YES: 8 (5, 10)	1.8	464	0.574	ns

Figure S4. PSA of all patients, grouped as 1. without prostate biopsy, 2. without csigPCa on subsequent biopsy and 3. with csigPCa on biopsy. Clinically significant prostate cancer (csigPCa) as defined as overall Gleason ≥3+4 of any length OR maximum cancer core length (MCCL) ≥4mm of any grade. Stratified by Likert score. Raw values for each patient shown as data points overlaying a boxplot displaying central line as median, lower and upper hinges as 25th and 75th percentiles (Q1 and Q3) and upper and lower whiskers as the last value no further than 1.5 * IQR from the hinge. Points coloured by patient age group and horizontal lines grey dashed lines at PSA values of 3 and 5 ng/ml. (Asterix denotes significance level in testing; * = P ≤ 0.05, ** = P ≤ 0.01, *** = P ≤ 0.001, **** =P ≤ 0.0001). Full statistical output in table below. Not all groups compared.

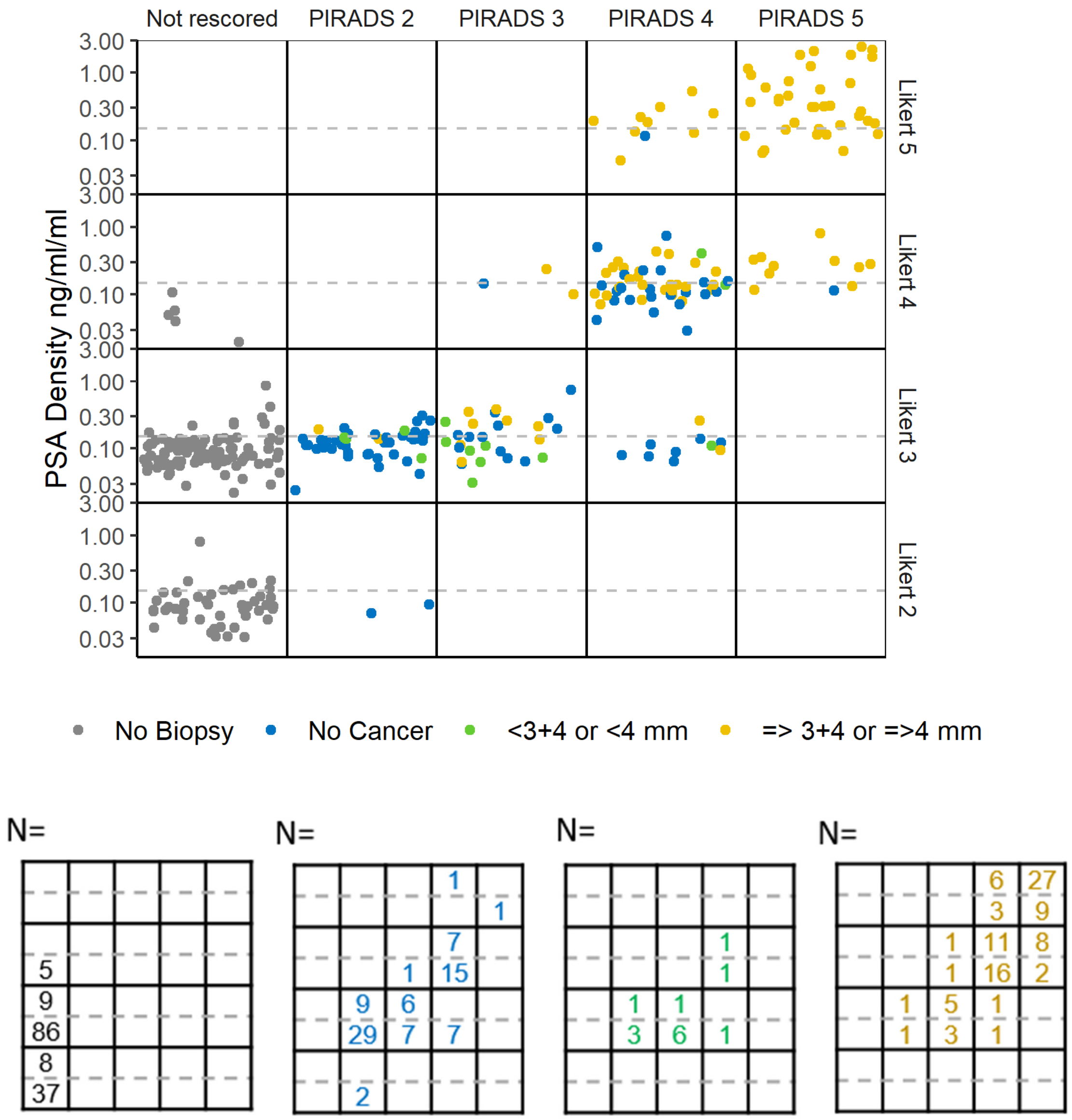

Figure S5. PSA density values for men plotted separately across their Likert and PI-RADS scores. Data points coloured as Figure 3. of any length or maximum cancer core length (MCCL) ≥4mm of any grade. Dashed line at PSAD = 0.15 ng/ml/ml for each group. Small tables show the numerical counts for each group, laid out to match the graph above, for this data the PSAD threshold was 0.15. PI-RADS was not used in the decision to biopsy and scored on in biopsied men retrospectively.

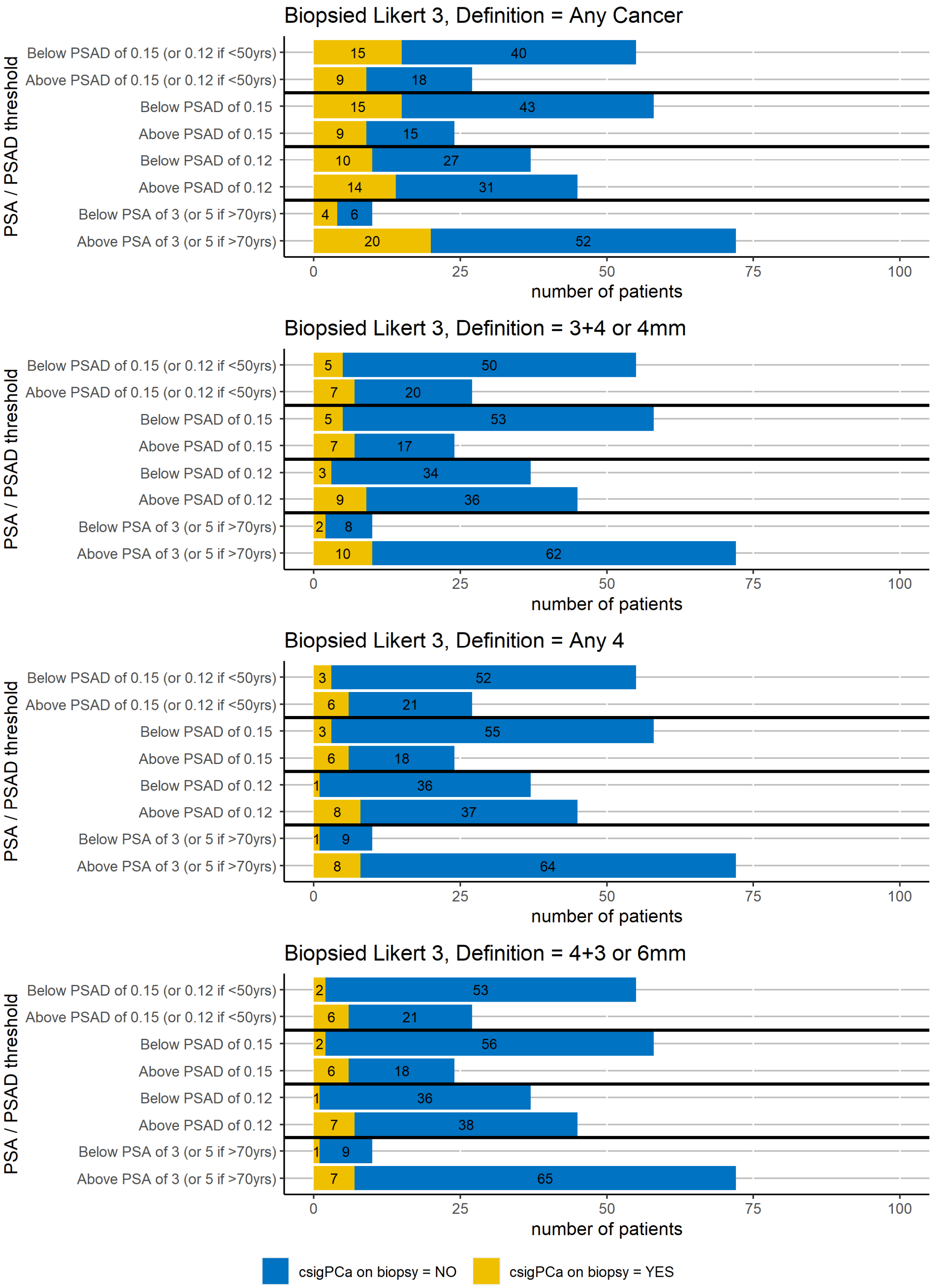

Figure S6. Only patients scored as Likert 3 who underwent prostate biopsy. Men grouped into presence of clinically significant prostate cancer on biopsy NO or YES using various definitions. Number of men in each group, stratified by ebign above or below 4 different threshold of PSA or PSAD.

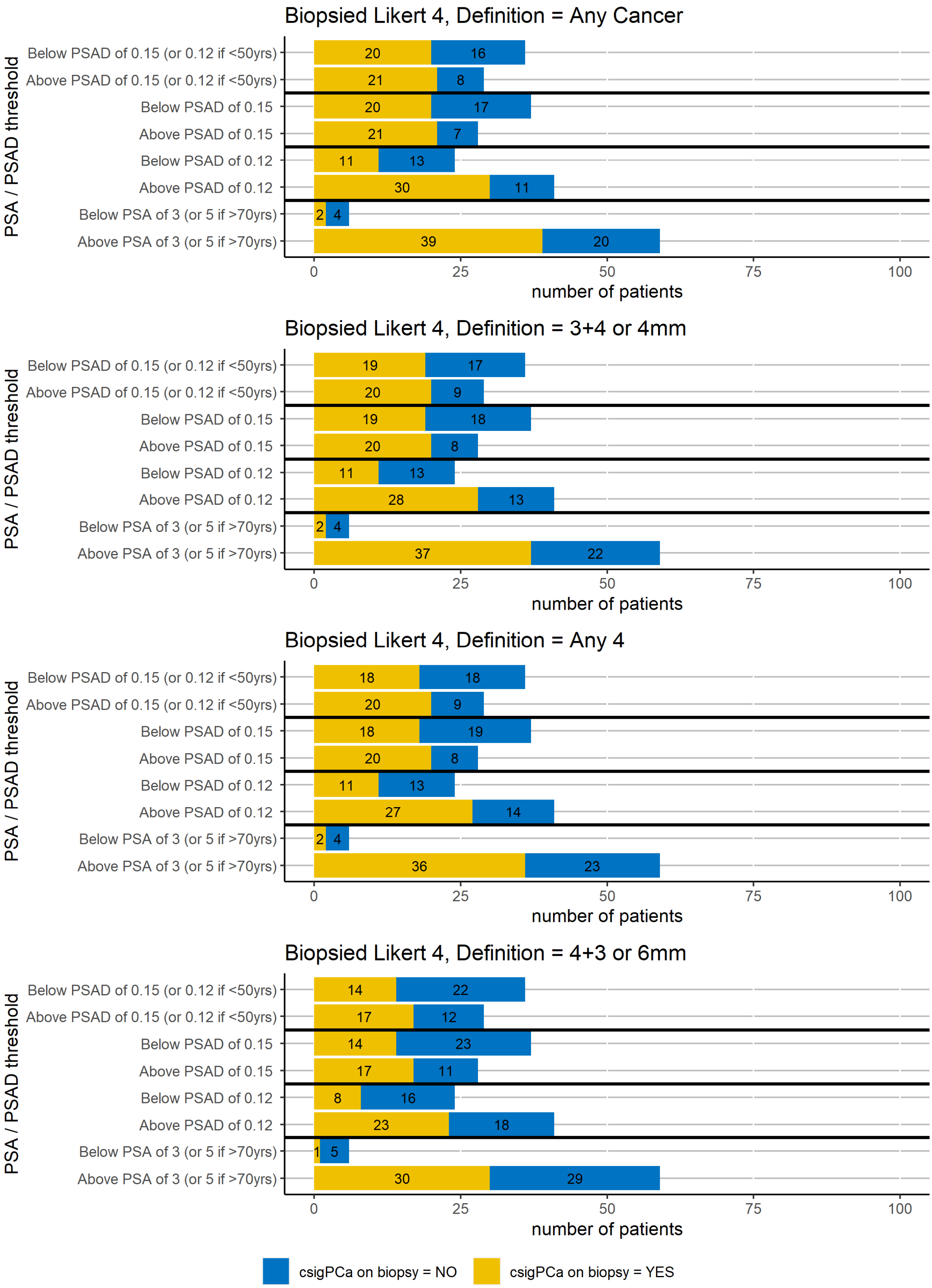

Figure S7. Only patients scored as Likert 4 who underwent prostate biopsy. Men grouped into presence of clinically significant prostate cancer on biopsy NO or YES using various definitions. Number of men in each group, stratified by ebign above or below 4 different threshold of PSA or PSAD.

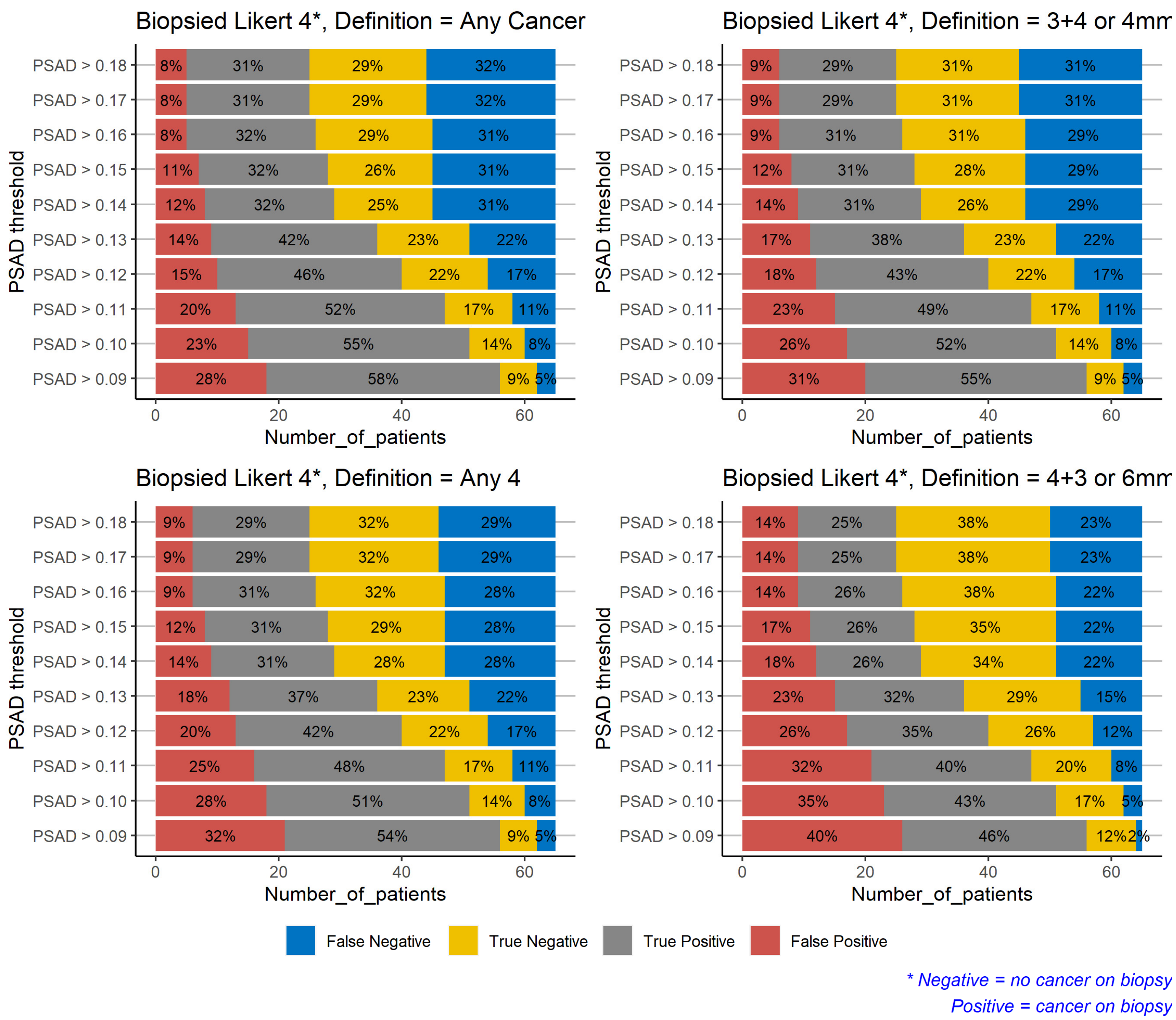

Figure S8. Only patients scored as Likert 4 who underwent prostate biopsy. A range of thresholds of PSAD used to predict the presence of clinically significant prostate cancer on biopsy using various definitions. Confusion matrix outputs; False and True Negative and False and True Positive as a percentage. Number of patients in each group readable using X axis.

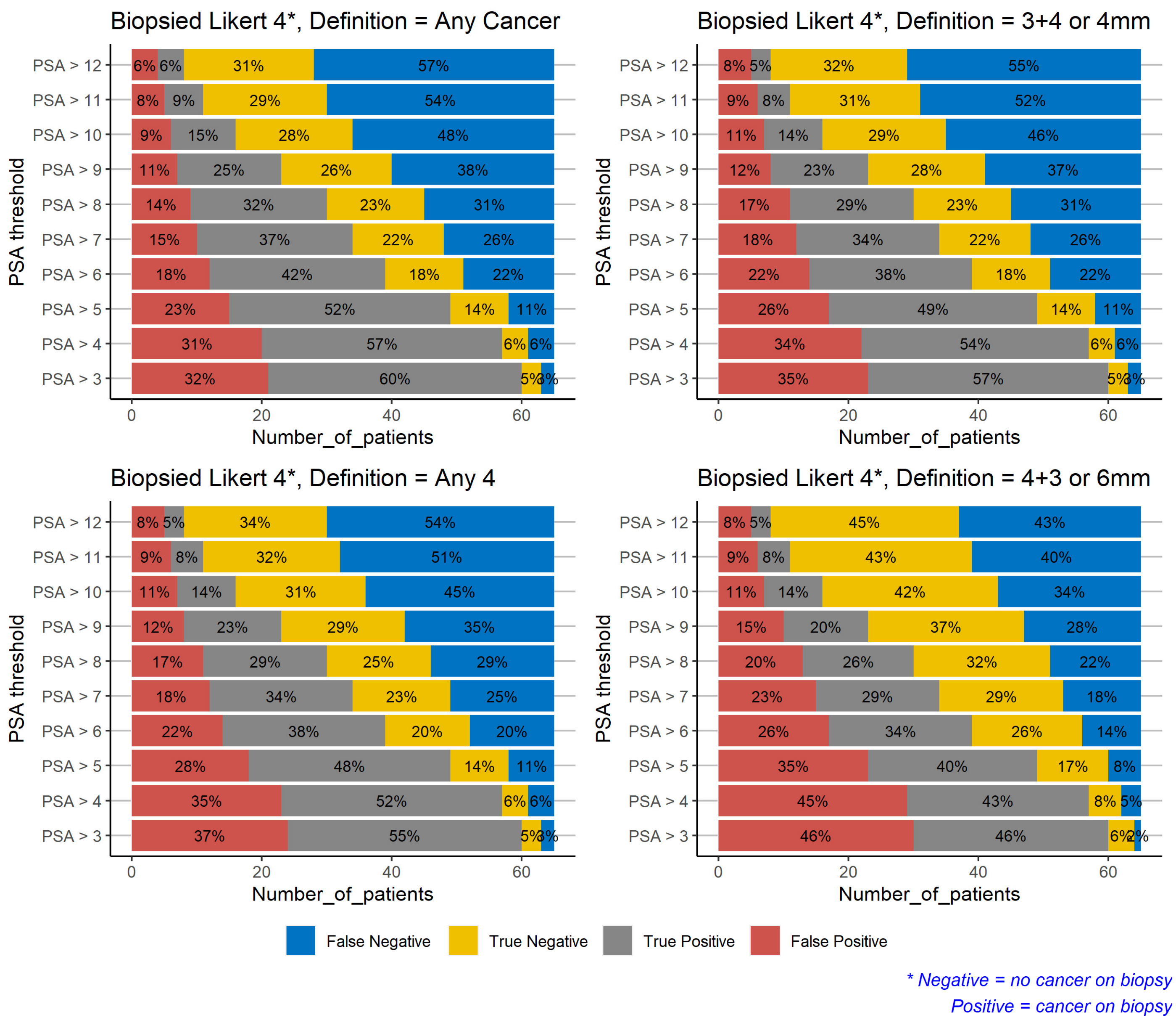

Figure S9. Only patients scored as Likert 4 who underwent prostate biopsy. A range of thresholds of PSA used to predict the presence of clinically significant prostate cancer on biopsy using various definitions. Confusion matrix outputs; False and True Negative and False and True Positive as a percentage. Number of patients in each group readable using X axis.

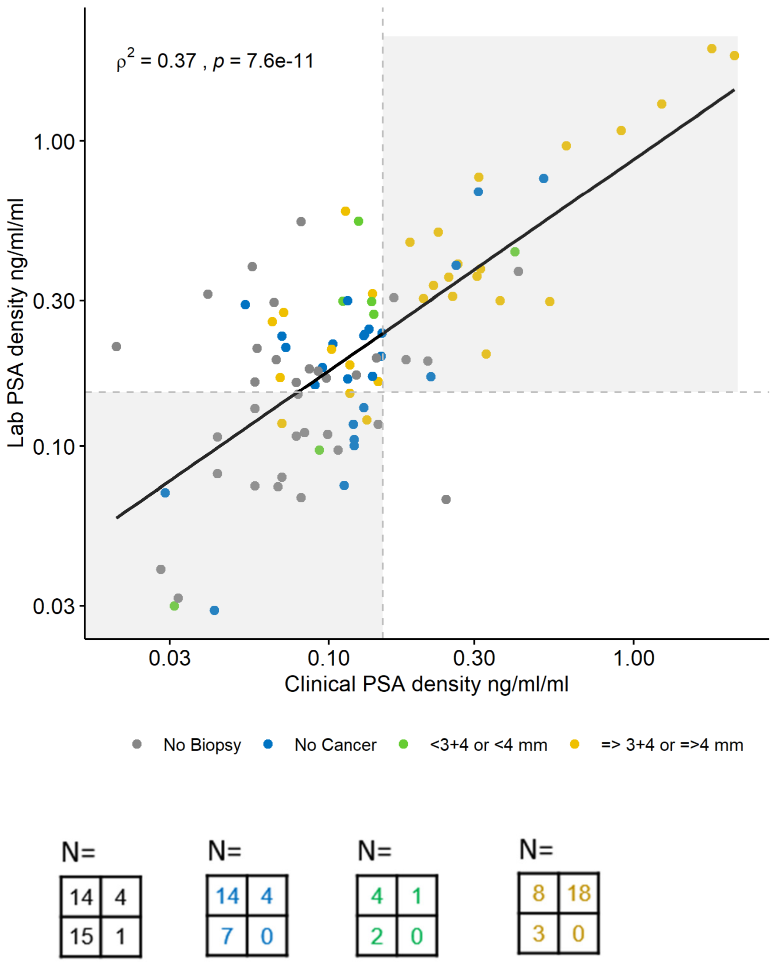

Characteristic	Both values below 0.15 N = 27 (28%)[1]	Both values above 0.15 N = 27 (28%)[1]	Re classified below 0.15 N = 1 (1.1%)[1]	Re classified above 0.15 N = 40 (42%)[1]
No Biopsy	15 (56%)	4 (15%)	1 (100%)	14 (35%)
No Cancer	7 (26%)	4 (15%)	0 (0%)	14 (35%)
Any Cancer	2 (7.4%)	1 (3.7%)	0 (0%)	4 (10%)
3+4 or 4mm	3 (11%)	18 (67%)	0 (0%)	8 (20%)

[1] Statistics presented: n (%)

Figure S10. Total serum PSA re-tested in 95 patients and converted to PSA density using the prostate volume as measured on mpMRI. Correlation between this and the reported 'clinical' PSA Density from patient notes. New serum sample taken at mpMRI, processed in our lab and sent for analysis at a clinical testing centre in Cambridge (CBAL). Displayed with linear regression line. Table showing the numbers of patients that would be classified above or below a PSAD threshold of 0.15 ng/ml/ml with both the 'clinical' PSA and the 'lab' PSA, alongside biopsy outcome.

Table S1. Further information about the prostate biopsies performed in this cohort. Statistics presented: Median (IQR) or n. Both centres take biopsies using trans rectal ultrasound guidance, and use a cognitive targeting method where targeted biopsies were taken. Biopsies done at UCLH are all done via the transperineal route and systematic sampling (if carried out) was taken from 12 Zone mapping (left anterior apex, left anterior base, right anterior apex, right anterior base, midline apex, midline base, left posterior apex, left posterior base, right posterior apex, right posterior base, left lateral, right lateral). Biopsies done at Barts Health are done via the transrectal route and systematic sampling (if carried out) was taken from either Ginsberg 6 zone mapping (n=17) (Zones; left anterior, left mid, left posterior, right posterior, right mid, right anterior) Ginsberg 8 zone mapping (n=2) (Zones; left anterior, left mid, left posterior, left base, right anterior, right mid ,right posterior, right base) or TRUS sextant (n=17) (Zones; right apex, right mid, right base, left apex, left mid, left base).

**Characteristic**	**Overall, N = 195**	**Barts Health N = 37**	**UCLH N = 158**
Age	65 (59, 70)	62 (58, 69)	65 (60, 70)
Referral PSA (ng/ml)	6.8 (5.0, 9.7)	7.8 (5.9, 11.3)	6.7 (4.6, 9.5)
PSA Density (ng/ml/ml)	0.14 (0.10, 0.25)	0.17 (0.12, 0.36)	0.14 (0.10, 0.23)
mpMRI prostate volume (ml)	45 (32, 62)	41 (31, 59)	46 (33, 63)
**MRI Highest Score (Likert)**			
2	2	0	2
3	82	13	69
4	65	10	55
5	46	14	32
**Biopsy description**			
Transperineal - Systematic sampling cores only	1	0	1
Transperineal - Both systematic sampling plus MRI-guided deployments	1	0	1
Transperineal - MRI guided deployment only	156	0	156
Transrectal - Systematic sampling cores only	8	8	0
Transrectal - Systematic sampling plus MRI-guided deployments	28	28	0
Transrectal - MRI guided deployment only	1	1	0
**Biopsy total number of cores**	14 (10, 18)	18.5 (16, 26)	13 (10, 17)
**Biopsy number of cores with cancer of any grade.**	2 (0, 5)	5 (1.5, 9)	1 (0, 5)
**Biopsy Overall Gleason Grade**			
5+5	1	1	0
5+4	2	1	1
5+3	0	0	0
4+5	3	0	3
3+5	2	0	2
4+4	4	2	2
4+3	19	5	14
3+4	61	11	50
3+3	18	5	13
Negative Biopsy	85	12	73
**Biopsy MCCL (mm)**	8.0 (4.0, 11.0)	10.5 (5.0, 13.2)	8.0 (4.0, 10.0)

Table S2. Proportion of patients above and below some clinically used PSA and PSAD thresholds. Grouped by (Table A) decision to biopsy and (Table B) presence of clinically significant prostate cancer on biopsy.

Table S2 (A)

Pre-biopsy mpMRI :	Patients did NOT have a biopsy (n=145)				Patients had a biopsy (n=195)			
	Likert 2, n = 45	Likert 3, n = 95	Likert 4, n = 5	Likert 5, n = 0	Likert 2, n = 2	Likert 3, n = 82	Likert 4, n = 65	Likert 5, n = 46
**PSA of 3 ng/ml (or 5ng/ml if over 70)**								
Above	36 (34%)	66 (62%)	4 (3.8%)	0 (0%)	2 (1.1%)	72 (41%)	59 (33%)	44 (25%)
Below	9 (23%)	29 (74%)	1 (2.6%)	0 (0%)	0 (0%)	10 (56%)	6 (33%)	2 (11%)
**PSAD of 0.15 ng/ml/ml**								
Above	8 (47%)	9 (53%)	0 (0%)	0 (0%)	0 (0%)	24 (28%)	28 (33%)	33 (39%)
Below	37 (29%)	86 (67%)	5 (3.9%)	0 (0%)	2 (1.8%)	58 (53%)	37 (34%)	13 (12%)
**PSAD of 0.12 ng/ml/ml**								
Above	13 (35%)	24 (65%)	0 (0%)	0 (0%)	0 (0%)	45 (36%)	41 (33%)	40 (32%)
Below	32 (30%)	71 (66%)	5 (4.6%)	0 (0%)	2 (2.9%)	37 (54%)	24 (35%)	6 (8.7%)
**PSAD of 0.15 ng/ml/ml (or 0.12ng/ml/ml if below 50*)**								
Above	8 (42%)	11 (58%)	0 (0%)	0 (0%)	0 (0%)	27 (30%)	29 (33%)	33 (37%)
Below	37 (29%)	84 (67%)	5 (4.0%)	0 (0%)	2 (1.9%)	55 (52%)	36 (34%)	13 (12%)

Statistics presented: n (% of row within group biopsy yes / no)

Table S2 (B)

Pre-biopsy mpMRI :	Patients who did NOT have clinically significant prostate cancer on biopsy (n=99)				Patients who had clinically significant prostate cancer on biopsy (n=96)			
	Likert 2, n = 2	Likert 3, n = 70	Likert 4, n = 26	Likert 5, n = 1	Likert 2, n = 0	Likert 3, n = 12	Likert 4, n = 39	Likert 5, n = 45
**PSA of 3 ng/ml (or 5ng/ml if over 70)**								
Above	2 (2.3%)	62 (71%)	22 (25%)	1 (1.1%)	0 (0%)	10 (11%)	37 (41%)	43 (48%)
Below	0 (0%)	8 (67%)	4 (33%)	0 (0%)	0 (0%)	2 (33%)	2 (33%)	2 (33%)
**PSAD of 0.15 ng/ml/ml**								
Above	0 (0%)	17 (68%)	8 (32%)	0 (0%)	0 (0%)	7 (12%)	20 (33%)	33 (55%)
Below	2 (2.7%)	53 (72%)	18 (24%)	1 (1.4%)	0 (0%)	5 (14%)	19 (53%)	12 (33%)
**PSAD of 0.12 ng/ml/ml**								
Above	0 (0%)	36 (73%)	13 (27%)	0 (0%)	0 (0%)	9 (12%)	28 (36%)	40 (52%)
Below	2 (4.0%)	34 (68%)	13 (26%)	1 (2.0%)	0 (0%)	3 (16%)	11 (58%)	5 (26%)
**PSAD of 0.15 ng/ml/ml (or 0.12ng/ml/ml if below 50)**								
Above	0 (0%)	20 (69%)	9 (31%)	0 (0%)	0 (0%)	7 (12%)	20 (33%)	33 (55%)
Below	2 (2.9%)	50 (71%)	17 (24%)	1 (1.4%)	0 (0%)	5 (14%)	19 (53%)	12 (33%)

Statistics presented: n (% of row within group csigPCa yes / no)

Supplementary text S1: Clinical outcome for Likert 2 and 5

Likert 2

In our cohort, only 2 men with Likert 2 (2/47) underwent biopsy, both showed no cancer of any grade (Table 1). The PSAD (PSA) values for these patients were 0.1 and 0.07 (4.19 and 5.46), these PSAD values are around the median (IQR) for all Likert 2 men; 0.09 (0.06-0.12). The full distribution of PSAD values for all men with Likert 2 can be seen graphically in (Figure S3, S4 and S5). When rescored with PI-RADS both were PI-RADS 2 lesions. Due to the fact most men with Likert 2 disease are already spared biopsy, it is unlikely novel serum or urine biomarkers could add any value here.

Likert 5

All men with Likert 5 (46/46) underwent biopsy, the median PSAD value for this group was 0.29 and all but one had csigPCa on subsequent biopsy (Table 1). This man had no cancer of any grade and a PSAD (PSA) of 0.114 (6.48). This value is in the bottom quartile for PSAD for a man with Likert 5 disease but by no means the lowest or an outlier for group as a whole. PSAD values for all men with Likert 5 can be seen graphically in (Figure S3, S4 and S5). Rescoring of all of the Likert 5 lesions with PI-RADS resulted in 22% (10/46) being re-classified with a lower PI-RADS score of 4, including the patient with no cancer on biopsy, no patients were downgraded to PI-RADS 2 or 3. Conversely 17% (11/65) of the rescored Likert 4 lesions were reclassified as a PI-RADS 5; of these again only 1 man had no cancer of any grade on biopsy, his PSAD (PSA) was 0.114 (6.14). There is unlikely to be space for novel serum and urine biomarkers to select Likert 5 or PIRADS 5 men for biopsy due to the high likelihood of csigPCa on biopsy.
